# Roles of Negatively Charged Histone Lysine Acylations in Regulating Nucleosome Structure and Dynamics

**DOI:** 10.3389/fmolb.2022.899013

**Published:** 2022-04-25

**Authors:** Yihang Jing, Xin Li, Zheng Liu, Xiang David Li

**Affiliations:** ^1^ Greater Bay Biomedical InnoCenter, Shenzhen Bay Laboratory (SZBL), Shenzhen, China; ^2^ Department of Chemistry, The University of Hong Kong, Hong Kong, China

**Keywords:** histone posttranslational modifications, lysine malonylation, lysine succinylation, lysine glutarylation, nucleosome structure, chromatin dynamics

## Abstract

The nucleosome, the basic repeating unit of chromatin, is a dynamic structure that consists of DNA and histones. Insights derived from biochemical and biophysical approaches have revealed that histones posttranslational modifications (PTMs) are key regulators of nucleosome structure and dynamics. Mounting evidence suggests that the newly identified negatively charged histone lysine acylations play significant roles in altering nucleosome and chromatin dynamics, subsequently affecting downstream DNA-templated processes including gene transcription and DNA damage repair. Here, we present an overview of the dynamic changes of nucleosome and chromatin structures in response to negatively charged histone lysine acylations, including lysine malonylation, lysine succinylation, and lysine glutarylation.

## Introduction

Eukaryotic cell nucleus contains chromatin structures that appear as “beads on a string” when observed under an electron microscope (Woodcock et al., 1976). The “beads” are nucleosomes that are the basic building blocks of chromatin, whereas the “string” contains the genomic DNA (Kornberg, 1974). The first high-resolution crystal structure of nucleosome was reported in 1997, which showed the nucleosome is composed of a histone octamer formed by one histone (H3-H4)_2_ tetramer and two histone H2A-H2B dimers wrapped by ∼147 base pairs of duplex DNA (Luger et al., 1997). The nucleosome is compacted and stabilized by intrinsic histone-histone and DNA-histone interactions. The nucleosome structure is highly dynamic and controls DNA accessibility, thus regulating all DNA-based physiological processes, such as replication, transcription, and DNA repair (Hauer and Gasser, 2017; Lai and Pugh, 2017). Over the past two decades, research has demonstrated that the nucleosome structure and dynamics can be regulated in several ways, including through covalent modifications of histones and DNA (Kouzarides, 2007; Zentner and Henikoff, 2013), incorporation of noncanonical histone variants (Talbert and Henikoff, 2010), and modulation by histone chaperones and nucleosome remodeling complexes (Hargreaves and Crabtree, 2011).

The flexible tails and the globular domain of histone can be decorated by various PTMs, ranging from the canonical PTMs such as lysine acetylation (Phillips, 1963), lysine methylation (Murray, 1964), and phosphorylation (Gutierrez and Hnilica, 1967) to more recently identified lysine crotonylation (Tan et al., 2011), lysine benzoylation (Huang et al., 2018), and lysine lactylation (Zhang et al., 2019). These histone PTMs can regulate nucleosome structure and dynamics by directly altering histone-histone or DNA-histone interactions, or serving as binding platforms for the recruitment of chromatin modifiers or remodeling enzymes (Bowman and Poirier, 2015). Since its identification in 1963, lysine acetylation has been well investigated. Early research unraveled a positive correlation between histone hyperacetylation and gene transcription activation (Allfrey et al., 1964). In the past 60 or so years, extensive research has revealed that lysine acetylation renders the chromatin to be in a more open state that enhances DNA accessibility (Zentner and Henikoff, 2013; Tessarz and Kouzarides, 2014), suggesting its positive role in gene transcription (Lai and Pugh, 2017). Indeed, from a chemistry perspective, acetylation neutralizes the positive charge on the ε-amino group of the lysine residues, which may compromise the DNA-histone or histone-histone interactions leading to increased DNA unwrapping (Neumann et al., 2009; Shimko et al., 2011) or facilitating nucleosome disassembly (Ye et al., 2005; Simon et al., 2011). In addition to the direct effects on nucleosome structures, it has been reported that lysine acetylation acts as an epigenetic signpost that is specifically recognized by chromatin readers, such as bromodomain-containing transcription factors, which then further recruit SWI/SNF chromatin remodelers that change the nucleosome structure and dynamics (Hassan et al., 2001; Carey et al., 2006; Ferreira et al., 2007; Chatterjee et al., 2011).

**FIGURE 1:**
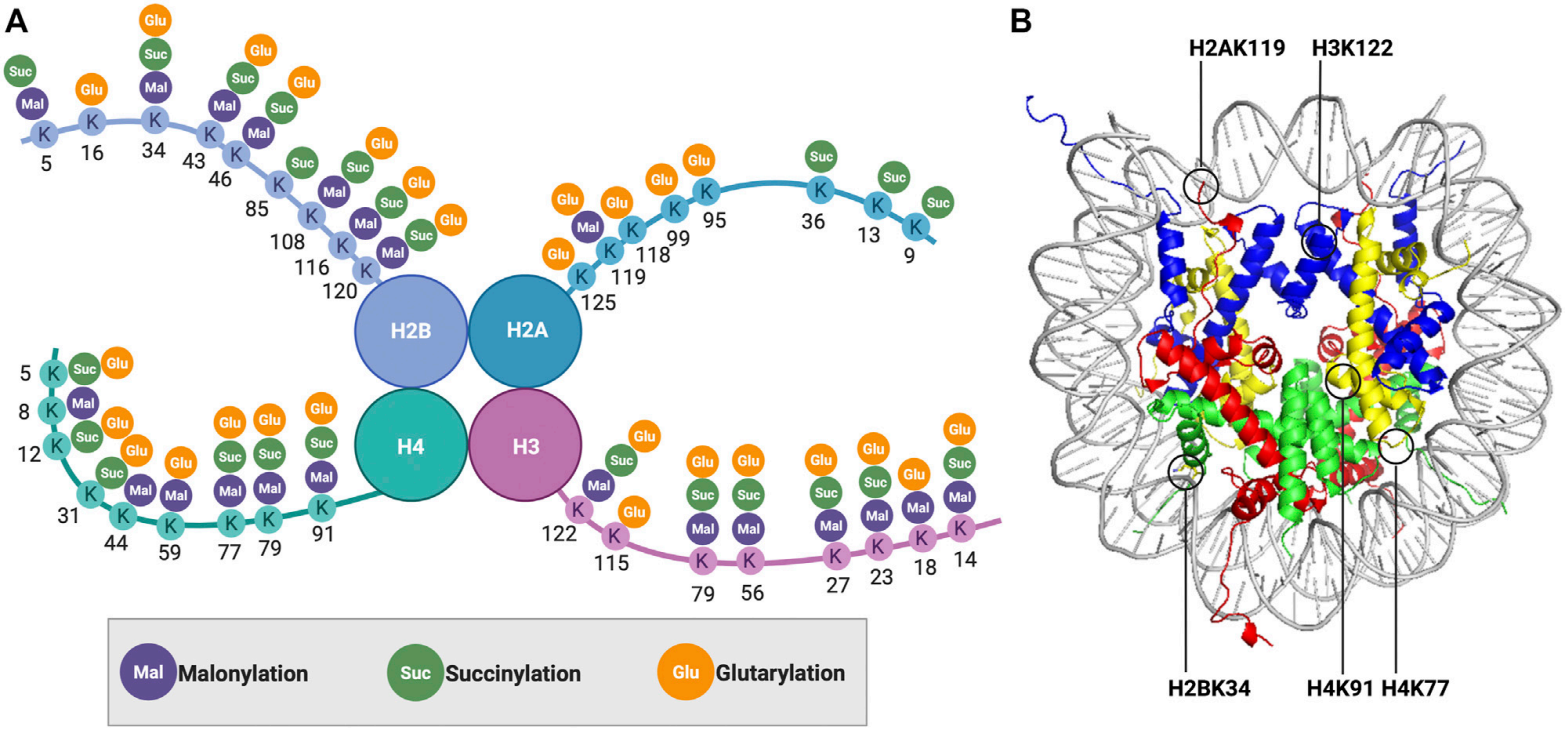
**(A)** Distribution of lysine malonylation, lysine succinylation, and lysine glutarylation on histones. **(B)** Nucleosomal localization of the modified histone lysine residues discussed in this mini-review.

With advances in mass spectrometry techniques, a series of new histone PTMs with special chemical properties were subsequently identified, including lysine malonylation (Xie et al., 2012), lysine succinylation (Xie et al., 2012), and lysine glutarylation (Bao et al., 2019) (Figure 1A). Compared with acetylation, besides increased acyl chain length, these acidic modifications also turn the positively charged lysine into negatively charged, which may lead to greater disturbances to the DNA-histone and histone-histone interactions. In this mini-review, we will discuss the current understanding of the influence of these negatively charged histone lysine acylations on nucleosome structure and dynamics. We also discuss potential future directions in this field. In the future, a better understanding of the regulatory mechanism is expected to provide new insights on the biological significance of these modifications.

## Role of Lysine Malonylation

Lysine malonylation was originally discovered on non-histone proteins (Peng et al., 2011) and was subsequently also identified on histones (Xie et al., 2012). Their pioneering work also demonstrated that, Sirt5, a member of the class III lysine deacetylases, can function as a demalonylase both *in vitro* and *in vivo* (Peng et al., 2011). Proteomic profiling revealed that most of the lysine malonylation substrates are metabolic enzymes. Biochemical studies confirmed the role of lysine malonylation in diverse metabolic pathways, including mitochondrial respiration (Colak et al., 2015), fatty acid metabolism (Hirschey and Zhao, 2015), and glycolysis (Nishida et al., 2015). In 2013, our lab was the first to develop MalAM-yne (Bao et al., 2013), a chemical reporter for lysine malonylation. This reporter can be used in the proteomic profiling of malonylated protein substrates in a variety of cells to further our understanding of the biological functions of lysine malonylation.

Under physiological conditions, the carboxylate group of malonyl changes the target lysine residue to a negative charge in a process similar to protein phosphorylation. Given that histone phosphorylation has been demonstrated to affect nucleosome dynamics and remodeling (Oki et al., 2007; North et al., 2011; Brehove et al., 2015), we can reasonably speculate that acidic lysine malonylation may also function to alter chromatin structure and dynamics. Such a hypothesis was tested and reported in a study by Kawashima and coworkers in 2018, which found the malonylation at histone H2A lysine 119 (H2AK119mal) (Figure 1B) resulted in chromosome segregation defects during mitosis and meiosis through downregulating proximal histone phosphorylation (Ishiguro et al., 2018). They initially performed mutation studies in budding yeast, in which H2AK119 was mutated to glutamate/aspartate (H2AK119E/D) to mimic lysine malonylation. These mutants displayed mis-segregation of the chromosomes and impaired chromosomal localization of shugoshin (Sgo1) proteins. The observed phenotypes were also conserved in fission yeast containing the same H2AK119 mutants. Considering that Bub1-mediated phosphorylation of adjacent serine 121 (H2AS121ph) was reported to recruit Sgo1 proteins to enable proper chromosome segregation in eukaryotes (Kawashima et al., 2010), the authors proposed that the crosstalk between H2AK119mal and Bub1-catalyzed H2AS121ph regulates chromosome segregation. To test this hypothesis, they performed *in vitro* phosphorylation and pull-down assays using synthesized unmodified or K119-malonylated H2A C-tail peptides. They found the H2AK119mal inhibited Bub1-dependent histone H2A phosphorylation by disrupting the electrostatic interaction between histone H2AK119 residue and E929 residue of Bub1. Overall, this study proposed a model in which the lysine malonylation affects the binding partners of histone, instead of directly altering the nucleosome structure and stability, which then regulates other histone PTMs to control chromatin function and dynamics.

## Role of Lysine Succinylation

Chemically, lysine succinylation and lysine malonylation are similar PTMs, only differing by one carbon in the acyl chain, and the enzyme pocket of Sirt5 can also recognize succinylated lysine and catalyze the removal of succinylation from lysine residues (Du et al., 2011). Nevertheless, proteomic profiling has revealed that the lysine malonylome (Nishida et al., 2015) is typically distinct from the lysine succinylome (Rardin et al., 2013), suggesting that lysine succinylation and malonylation may have unique functions and regulatory patterns. Although succinylation can be installed non-enzymatically using succinyl-CoA as a cofactor *in vitro*, it has been revealed that succinylation of H3K79 can be catalyzed by lysine acetyltransferase 2A (KAT2A, also known as GCN5) in cells to promote tumor cell proliferation and tumor development (Wang et al., 2017). Moreover, carnitine palmitoyltransferase (CPT) 1A was reported to function as a novel succinyltransferase both *in vivo* and *in vitro* (Kurmi et al., 2018). Very recently, histone acetyltransferase 1 (HAT1) was demonstrated to modulate lysine succinylation on various proteins including histones and non-histones, and HAT1 succinylates histone H3 on K122 (H3K122), which contributes to epigenetic regulation and gene expression in cancer cells (Yang et al., 2021). YEATS domains were identified as epigenetic readers of histone acetylation and crotonylation marks (Li et al., 2022). YEATS family proteins serve as members of chromatin-modifying and transcription complexes, participating in chromatin remodeling and transcriptional regulation (Schulze et al., 2009). The human genome encodes four YEATS domain-containing proteins, ENL, AF9, YEATS2, and GAS41. In 2018, Hao and coworkers identified the YEATS domain of GAS41 as the first reader of histone succinylation in a pH-dependent manner (Wang et al., 2018). Biochemical assays confirmed the significant binding affinity of the GAS41 YEATS domain toward succinylation of histone H3 at lysine 122 (H3K122succ). Co-crystal structure of the YEATS domain of Yaf9, the GAS41 homolog, in complex with an H3K122succ peptide demonstrated that the carboxyl terminal of the succinyl group will form a salt bridge with a protonated histidine (His39) residue of the YEATS domain under acidic pH environment.

**FIGURE 2:**
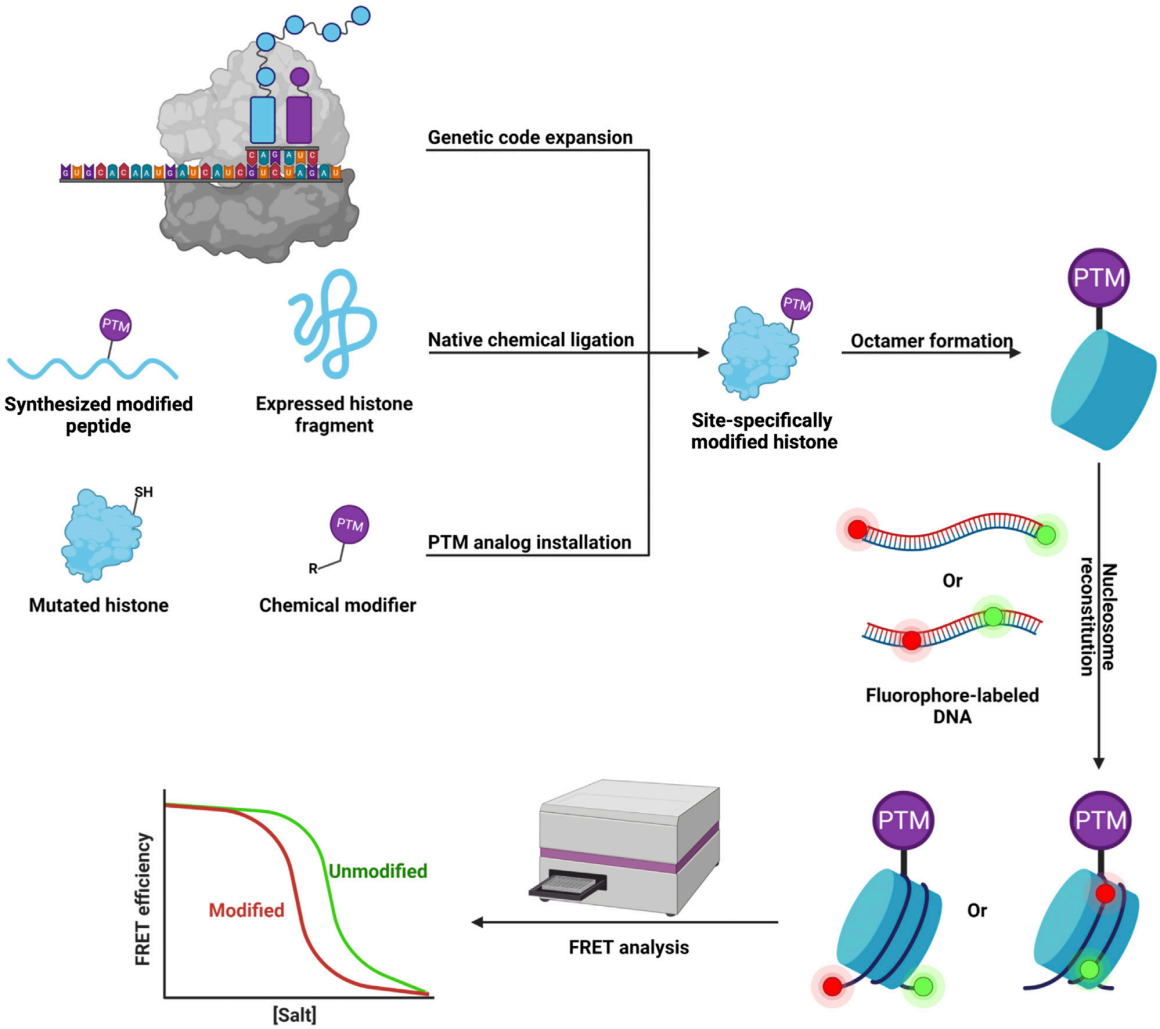
Workflow of the *in vitro* FRET-based assay for studying the effects of site-specific histone PTM on nucleosome dynamics.

To unravel the influence of lysine succinylation on nucleosome structure and dynamics, our group examined histone H2B succinylation at lysine 34 (Jing et al., 2018) (Figure 1B). We first developed a robust two-step chemical approach that allows the rapid incorporation of a succinyl lysine analog (K_c_succ) into specific residues on the histone. Biochemical analysis showed that the K_c_succ could be recognized by an anti-succinyl lysine antibody as well as by Sirt5, indicating our succinylation mimic had high structural and functional similarity to its native counterpart. Next, we utilized this approach to introduce K_c_succ into histone H2B at lysine 34 (H2BK34), a succinylation site located at the DNA-histone interface within the nucleosome. Using a fluorescence resonance energy transfer (FRET)-based nucleosome disassembly assay (Figure 2), we were the first to demonstrate that succinylation at H2BK34 decreased nucleosome stability and altered its dynamics. Given that nucleosomes disassemble in a stepwise manner with increasing ionic strength (Bohm et al., 2011), we put two fluorophores at different positions on the nucleosome to monitor the dynamic disassembly processes in real time using the FRET-based assay. In one FRET experiment, two fluorophores were placed at the two ends of the DNA to evaluate the opening of the nucleosome and release of the H2A-H2B dimer from the DNA. In another FRET experiment, two fluorophores were placed in the middle of the DNA to monitor the final dissociation of (H3-H4)_2_ tetramer from the nucleosome. The FRET results revealed that succinylation of H2BK34 promoted nucleosomal unfolding, leading to the release of the histone H2A-H2B dimers, likely through the direct effect of the succinylation on DNA-histone interaction, while there was no impact on the subsequent histone (H3-H4)_2_ tetramer dissociation. The FRET results were consistent with defects in the chromatin structure of a budding yeast strain containing a lysine-to-glutamate (K-to-E) mutation at the corresponding residue of yeast histone H2B. Collectively, our pioneering work provides a simple method for the rapid generation of recombinant histones with site-specific succinylation mimics, which revealed the novel regulatory machine of histone succinylation on the dynamic organization of chromatin.

Intrigued by the universal effects on nucleosome and chromatin dynamics due to succinylation of lysine residues located at the DNA-histone interface, we further examined succinylation of histone H4 at lysine 77 (Jing et al., 2020) (Figure 1B). Here, we used the canonical expressed protein ligation (EPL) strategy (Muir et al., 1998) to prepare histone H4 with site-specific succinylation at the K77 residue (H4K77succ). Besides the DNA labeling strategies mentioned above, in this study, we prepared another set of FRET samples with the two fluorophores at histone H2A and one end of the DNA separately to have a more straightforward view of the dimer release. The single-molecule FRET analysis of these samples indicated that H4K77succ destabilized the nucleosome and promoted the release of the H2A-H2B dimers. Using a LexA binding-based FRET assay (North et al., 2012), we revealed that H4K77succ facilitated DNA unwrapping at the entry-exit region of the nucleosome to promote the release of the H2A-H2B dimers and increase DNA accessibility, which was also verified by our collaborators utilizing optical tweezer assays. Consistently, such destabilizing effects were also observed in budding yeast containing a K-to-E mutation at the corresponding residue. Likewise, a very recent study using a similar FRET approach showed the p300/CBP-dependent succinylation of H3K122 (Figure 1B), also located within the histone core, destabilized the nucleosome, which enhanced gene transcription as assessed by an *in vitro* transcription assay (Shahidian et al., 2021). A cellular function study of Sirt7 revealed that the Sirt7-catalyzed desuccinylation of H3K122 promoted chromatin condensation and DNA-damage repair (Li et al., 2016). Moreover, metabolically driven hypersuccinylation of histones was reported to weaken nucleosome stability (Smestad et al., 2020), further underlining the general effects of lysine succinylation on nucleosome dynamics.

## Role of Lysine Glutarylation

The discovery of lysine glutarylation in 2014 (Tan et al., 2014) further expands the landscape of acidic lysine acylations. Glutaryl-CoA is an important intermediate in amino acid metabolism and can function as a cofactor in this modification reaction. Proteome-wide profiling showed this epigenetic mark was enriched on metabolic enzymes and mitochondrial proteins, which supports that lysine glutarylation has significant functional roles in cellular regulation and diseases (Hirschey and Zhao, 2015; Sabari et al., 2017). Compared with histone lysine malonylation and succinylation, histone lysine glutarylation is presumed to affect nucleosome structure and dynamics to the greatest extent as it has the longest acyl chain. However, regulation of chromatin structure and dynamics by histone lysine glutarylation remains poorly understood.

Inspired by the identification of three glutarylated lysine residues in histone H2B(Tan et al., 2014), we developed GluAM-yne, a chemical reporter for lysine glutarylation, which we used to systematically map histone lysine glutarylation (Bao et al., 2019). We identified 27 histone lysine glutarylation sites, of which histone H4 lysine 91 (H4K91) was postulated to have the most potential impact due to its localization at the interface of histone dimer and tetramer (Figure 1B). Meanwhile, the mononucleosome structure showed that H4K91 forms a salt bridge with histone H2B glutamic acid 63 (H2BE63) (Cosgrove et al., 2004). Therefore, glutarylation at H4K91glu was speculated to regulate nucleosome structure and dynamics by affecting histone-histone interactions. To test such a hypothesis, we utilized the EPL strategy to prepare homogenous histone H4 with site-specific glutarylation stoichiometrically installed at the K91 residue and then reconstituted it into histone octamers *in vitro*. Elution profiles of H4K91glu-containing octamers and unmodified octamers by size-exclusion chromatography indicated that the octamer assembly was destabilized by H4K91glu, which was also validated by *in vitro* nucleosome reconstitution experiments. Furthermore, we used similar FRET-based approaches as above to demonstrate that H4K91glu promoted the dissociation of histone H2A-H2B dimers from mononucleosomes. *In vivo* studies in budding yeast showed that a mutation of H4K91 to glutamate mimicking the H4K91glu destabilized the chromatin structure, leading to global transcription upregulation and defects in DNA damage repair and cell cycle progression. More strikingly, although Sirt5 was reported as an *in vitro* deglutarylase (Tan et al., 2014), we used a site-specific anti-H4K91glu antibody to show that Sirt7 was the endogenous deglutarylase targeting H4K91glu *in vivo.* Moreover, Sirt7-catalyzed deglutarylation of H4K91glu promoted chromatin condensation in DNA damage repair. In summary, our studies showed, for the first time, the effects of site-specific glutarylation on the structure and dynamics of chromatin and provided mechanistic insights into the cellular roles of H4K91 glutarylation in gene transcription regulation and DNA damage response.

## Conclusion and Perspectives

The current research on negatively charged histone lysine acylations has demonstrated they all have impacts on nucleosome structure and dynamics, but with diverse molecular mechanisms. Like the interaction between the GAS41 YEATS domain and H3K122succ, we believe that the negative charge of these newly identified histone acylations may play significant roles in protein-protein interactions (PPIs) between these acidic histone PTMs and their interactomes. The interactomes of these newly identified histone acylations can be systematically profiled with the aid of chemical proteomics approaches (Li and Kapoor, 2010; Li et al., 2012; Bao et al., 2014), which is crucial for further understanding of the cellular functions of these acidic histone PTMs. Besides the direct effects on nucleosome architecture, future studies should also focus on the influence of acidic histone PTMs-mediated PPIs or possible crosstalk with other histone PTMs on the nucleosome structure and dynamics, like the example discussed in the malonylation part of this mini-review. These negatively charged lysine acylations have also been found on linker histone H1 (Huang et al., 2014; Sabari et al., 2017), which plays a significant role in maintaining the higher-order structure of chromatin (Finch and Klug, 1976). Recent studies have shown phosphorylation (Lopez et al., 2015) or acetylation (Li et al., 2018) of histone H1 can affect chromatin condensation. However, the effects of acidic lysine acylations of histone H1 on chromatin compaction are still unknown. In addition to histones, our recent research has expanded to investigate the effects of succinylation of non-histone chromosomal proteins on nucleosome dynamics (Jing et al., 2021). We found that the incorporation of the above-mentioned K_c_succ into the nucleosome-binding domain of non-histone chromosomal protein HMG-17 (HMGN2) weakened the electrostatic interaction between the nucleosome-binding domain and the acidic patch of the nucleosome, leading to the dissociation of HMGN2 from the nucleosome, which enhanced DNA unwrapping in the entry/exit region of nucleosome. These findings could serve to stimulate the complementary investigation of the acidic acylation of non-histone chromosomal proteins to advance our understanding of their functional roles in chromatin dynamics and related biological processes.

Lysine malonylation, succinylation, and glutarylation reactions all utilize the corresponding acyl-CoA as the cofactor donating the acyl group. These acyl-CoAs are important intermediates of cellular metabolism, e.g., malonyl-CoA can be synthesized *de novo* from glucose (Wolfgang et al., 2007), succinyl-CoA is an import intermediate in the TCA cycle (Ottaway et al., 1981), and glutaryl-CoA is involved in amino acid metabolism (LaRossa, 2013). Thus, these acyl-CoAs can be the bridges between cellular metabolism and the epigenome (Trefely et al., 2020), and research evidence has also endorsed the positive correlations between cellular levels of distinct acyl-CoAs and the abundance of corresponding histone acylations (Simithy et al., 2017). It has been documented that histones can be the sensors of cellular acetyl-CoA concentration to regulate chromatin structure and gene expression (Wellen et al., 2009; Pietrocola et al., 2015; Sivanand et al., 2018). Nevertheless, the metabolic regulation of chromatin dynamics involving these newly identified acidic lysine acylations and their roles in physiological processes and disease pathogenesis are still unclear, which could be the focus of future research.

It has been reported that aberrant acetylation or deacetylation are associated with the development and progression of cancers, human developmental disorders, and metabolic diseases (Timmermann et al., 2001; Iyer et al., 2012; Sheikh, 2014). This has prompted research focusing on the role of the negatively charged histone lysine acylations in disease pathogenesis. For example, Wang et al. revealed the succinylation of histone H3 on lysine 79 (H3K79succ) by acetyltransferase 2A (KAT2A, also known as Gcn5) was instrumental in tumor cell proliferation and tumor development (Wang et al., 2017). Furthermore, two very recent studies demonstrated the functional roles of KAT2A-dependent H3K79succ in human pancreatic ductal adenocarcinoma (Tong et al., 2020) and Hepatitis B virus (Yuan et al., 2020), respectively. Similarly, it was proposed that upregulated lysine malonylation level was responsible for the development of type 2 diabetes (Du et al., 2015). Moreover, the pathophysiological functions of lysine glutarylation have been unraveled in various diseases, such as asthenospermia (Cheng et al., 2019) and glutaric acidemia type 1 disease (Schmiesing et al., 2018). All these studies have demonstrated the functional significance of these negatively charged histone lysine acylations on the pathogenesis of human diseases, which warrants future research into these acidic histone PTMs as promising therapeutic targets.
